# Future in the Past: *Azorella glabra* Wedd. as a Source of New Natural Compounds with Antiproliferative and Cytotoxic Activity on Multiple Myeloma Cells

**DOI:** 10.3390/ijms19113348

**Published:** 2018-10-26

**Authors:** Daniela Lamorte, Immacolata Faraone, Ilaria Laurenzana, Luigi Milella, Stefania Trino, Luciana De Luca, Luigi Del Vecchio, Maria Francesca Armentano, Chiara Sinisgalli, Lucia Chiummiento, Daniela Russo, Faustino Bisaccia, Pellegrino Musto, Antonella Caivano

**Affiliations:** 1 Laboratory of Preclinical and Translational Research, IRCCS “Referral Cancer Center of Basilicata” (CROB), 85028 Rionero in Vulture, PZ, Italy; daniela.lamorte@crob.it (D.L.); stefania.trino@gmail.com (S.T.); luciana.deluca@crob.it (L.D.L.); antonella.caivano@crob.it (A.C.); 2 Department of Science, University of Basilicata, 85100 Potenza, Italy; immafaraone88@gmail.com (I.F.); mariafrancesca.armentano@unibas.it (M.F.A.); chiara.sinisgalli@gmail.com (C.S.); lucia.chiummiento@unibas.it (L.C.); daniela.russo@unibas.it (D.R.); faustino.bisaccia@unibas.it (F.B.); 3 Department of Molecular Medicine and Medical Biotechnology, Federico II University, 80131 Naples, Italy; 4 Hematology and Stem Cell Transplantation Unit, IRCCS “Referral Cancer Center of Basilicata” (CROB), 85028 Rionero in Vulture, PZ, Italy; pellegrino.musto@crob.it

**Keywords:** *Azorella glabra* Wedd., phytochemicals, multiple myeloma, cytotoxic effect, apoptosis, cell cycle arrest

## Abstract

Multiple myeloma (MM) is the second most common hematologic malignancy and, although the development of novel agents has improved survival of patients, to date, it remains incurable. Thus, newer and more effective therapeutic strategies against this malignancy are necessary. Plant extracts play an important role in anti-tumor drug discovery. For this reason, in the investigation of novel natural anti-MM agents, we evaluated the phytochemical profiles, in vitro antioxidant activity, and effects on MM cells of *Azorella glabra* (AG) Wedd. Total polyphenols (TPC), flavonoids (TFC), and terpenoids (TTeC) contents were different among samples and the richest fractions in polyphenols demonstrated a higher antioxidant activity in in vitro assays. Some fractions showed a dose and time dependent anti-proliferative activity on MM cells. The chloroform fraction (CHCl_3_) showed major effects in terms of reduction of cell viability, induction of apoptosis, and cell cycle arrest on MM cells. The apoptosis induction was also confirmed by the activation of caspase-3. Importantly, the CHCl_3_ fraction exhibited a negligible effect on the viability of healthy cells. These results encourage further investigations on AG extracts to identify specific bioactive compounds and to define their potential applications in MM.

## 1. Introduction

Multiple myeloma (MM) is still an incurable hematologic malignancy characterized by a clonal growth of plasma cells in the bone marrow [1]. MM is the second most frequent hematologic malignancy [2], with an incidence rate of 6.2 per 1 × 10^5^ individuals [3], and it mainly affects individuals with a median age of 65–70 years at diagnosis [4]. This neoplasm is associated with a five year overall survival of 48.5% [5]. Although hematopoietic stem cell transplantation and novel targeted agents, such as proteasome inhibitors [6,7], monoclonal antibodies [8,9], immunomodulatory drugs [10], check-point inhibitors [11], and epigenetic modulators [12], have significantly achieved lasting remission and increased survival rates [13], most patients relapse, develop resistance, and eventually die because of refractory disease [14]. All these issues highlight the need to investigate newer therapeutic targets [15,16] to improve patient outcomes.

Plant extracts play an important role as a new therapeutic strategy in cancer [2] because they include various types of metabolites with different chemical structures and bioactivities. In fact, by synergistic and/or additive effects [17,18,19] they target different pathways in malignant cells, such as proliferation, differentiation, and apoptosis [20]. Furthermore, plant extracts have a favorable profile of absorption and metabolism and show no or low toxicity towards normal cells. The biological activities of plant extracts are mainly due to their content in polyphenols, flavonoids, and terpenoids. Numerous studies showed that polyphenols, generally recognized as antioxidants, possess anticancer and pro-apoptosis properties [21,22]. Other studies reported the potential clinical applications of flavonoids for their well-known protective and therapeutic effects against cancer, cardiovascular, and neurodegenerative diseases [23], and of terpenoids, for their anti-cancer, anti-malarial, anti-inflammatory, anti-bacterial, and anti-viral activities [24].

The combination of plant extracts with anti-cancer drugs may offer a significant advantage for therapeutic efficacy by sensitizing malignant cells to drugs and overcoming drug-induced resistance in cancer [25]. For all these reasons, a significant number of compounds isolated from plants are still used nowadays in cancer clinical practice in combination with other drugs [26], also against hematologic malignancies [27,28,29,30,31].

During the last years, research has focused on novel plant extract metabolites as possible anti-tumor agents on various types of cancer, including hematologic malignancies; recent work demonstrated the cytotoxic effects of various species of *Centaurea* genus against various cancer cell lines, including a panel of hematologic malignancies cells, such as pre-B-ALL, MM, and acute promyelocytic leukemia (APL) [32]. Kabeel et al. (2018) showed the anti-leukemic effects of a mixture of four water plant extracts (*Arctium lappa*, *Ulmus rubra*, *Rumex acetosella*, and *Rheum palmatum*) in a leukemic rat model [33]. In addition, it was previously reported that *Azorella compacta* methanolic extract induced apoptosis in leukemia cells [34].

Furthermore, in the past decades, plant extracts have attracted much attention also in the field of MM. Shammas et al. (2006) demonstrated that epigallocatechin-3-gallate, an abundant polyphenol in green tea, possesses anti-MM effects in in vitro and in vivo assays [35]. More recently, Wang and colleagues (2015) found that aloperine, a natural alkaloid isolated from the herb, *Sophora alopecuroides*, inhibited MM cell growth in in vitro and in vivo assays; reduced bone lesions in a human MM xenograft mouse model, increasing their survival; and showed a synergistic inhibitory effect on MM growth with bortezomib [36]. In addition, the in vitro anti-MM effects of *Punica granatum* extracts were demonstrated [20].

In the present study, for the first time and to the best of our knowledge, the phytochemicals, the in vitro antioxidant properties, and the effects on MM cells of *Azorella glabra* (AG) aerial parts, a plant belonging to the Apiaceae family [37], have been investigated.

## 2. Results

### 2.1. Extraction Yield and Total Polyphenol (TPC), Flavonoid (TFC), and Terpenoid (TTeC) Content of AG Extract and Fractions

**Figure 1:**
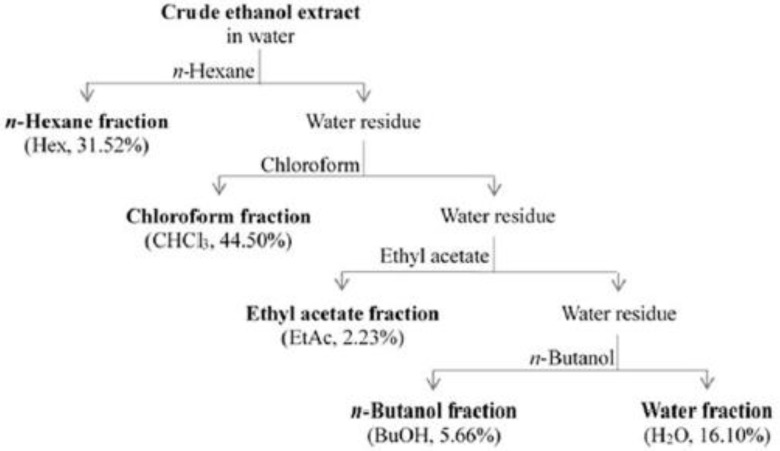
Schematic representation of liquid/liquid extraction of AG aerial parts.

The aerial parts of AG were dried at room temperature and extracted using 96% ethanol (EtOH) by dynamic maceration with an extraction yield of 9.01%. Then, the crude EtOH extract, named with acronym EtOH, was separated based on the affinity solvent by liquid/liquid extraction using an increasing solvent polarity obtaining the fractions named with following acronyms: Hex for *n*-hexane solvent, CHCl_3_ for chloroform solvent, EtAc for ethyl acetate solvent, BuOH for *n*-butanol solvent and H_2_O for water solvent. The Hex and CHCl_3_ fractions showed the highest extraction yield (44.50% and 31.52%, respectively); instead, the EtAc and BuOH fractions demonstrated the lower extraction yields (2.23% and 5.66%, respectively) (Figure 1).

**Figure 2:**
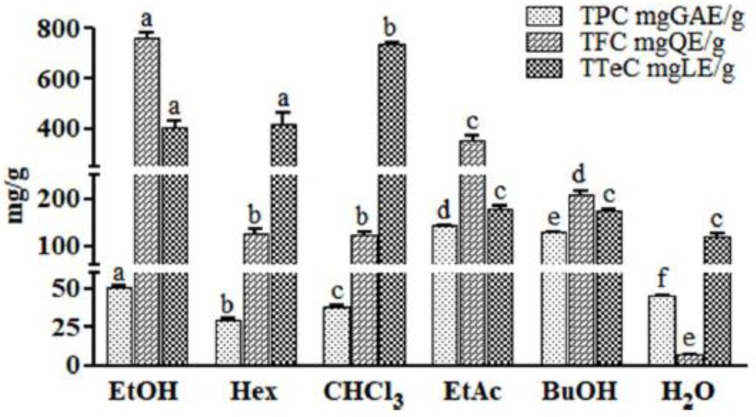
Total polyphenol content (TPC), total flavonoid content (TFC), and total terpenoid content (TTeC) of AG samples. Results were expressed as mean ± standard deviation of triplicate determinations in mg of gallic acid equivalents per gram of dried sample (mg GAE/g), in mg of quercetin equivalents per gram of dried sample (mg QE/g), and in mg of linalool equivalents per gram of dried sample (mg LE/g). For each test, the values with identical letters are not significantly different at the *p* < 0.05 level, 95% confidence limit, according to one-way analysis of variance (ANOVA). Samples are ethanol extract (EtOH) and *n*-hexane (Hex), chloroform (CHCl_3_), ethyl acetate (EtAc), *n*-butanol (BuOH), and water (H_2_O) fractions of AG aerial parts.

Three different in vitro colorimetric methods were used for the determination of the phytochemical profiles of each fraction in terms of TPC, TFC, and TTeC (Figure 2). Samples displayed quantitative differences in TPC value, with a mean value of 72.52 mg of gallic acid equivalents per gram of dried sample (mg GAE/g). The EtAc and BuOH fractions showed a higher TPC than other fractions (143.60 ± 0.40 and 128.14 ± 0.74 mg GAE/g, respectively). In addition, flavonoids, a class of polyphenols with several biological properties [38], have been measured in AG samples. TFC showed a mean value of 264.13 mg of quercetin equivalents per gram of dried sample (mgQE/g). The EtOH extract, the EtAc, and BuOH fractions showed higher TFC (764.86 ± 16.76, 354.97 ± 22.05, and 209.73 ± 5.56 mg QE/g, respectively) than other fractions. Moreover, TTeC was also determined. The CHCl_3_ and Hex fractions and the EtOH extract exhibited higher values (733.51 ± 9.42, 421.77 ± 41.46, and 405.44 ± 29.33 mg LE/g, respectively) than the mean value of 338.38 mg of linalool equivalents per gram of dried sample (mg LE/g).

### 2.2. Radical-Scavenging Activity

The antioxidant activity of AG samples was tested by the determination of the radical scavenging activity by synthetic 2.2′-azino-bis(3-ethylbenzothiazoline-6-sulfonic acid) (ABTS) and physiological superoxide anion (SO) and nitric oxide (NO) radicals (Table 1).

The EtAc fraction showed the highest radical scavenging-activity in the ABTS assay with a value of 282.26 ± 9.53 mg TE/g, followed by the BuOH fraction; instead, the Hex fraction was inactive.

The ability of samples to scavenge biological SO and NO was expressed as IC_25_, and results were compared with ascorbic acid. All samples, except for Hex fraction, caused a dose-dependent inhibition in the SO assay. In particular, the EtAc and BuOH fractions showed higher activity (IC_25_ of 0.12 ± 0.01 and 0.20 ± 0.01 mg/mL, respectively) than ascorbic acid (IC_25_ of 0.26 ± 0.02 mg/mL). The scavenging ability against NO was only reported by the BuOH and H_2_O fractions, with a dose-dependent inhibition with higher values of ascorbic acid (IC_25_ 4.78 ± 0.09 mg/mL).

The linear correlation coefficient (r) was calculated by the Pearson test. The results showed a strong correlation between polyphenols and antioxidant activity. In fact, the highest correlation was found between the TPC and radical-scavenging activity against ABTS and super oxide radicals (r_TPC/ABTS_ = 0.99 and r_TPC/SO_ = 0.92). Instead, the terpenoids were less involved in the scavenging-activity (r < 0).

**Figure 3:**
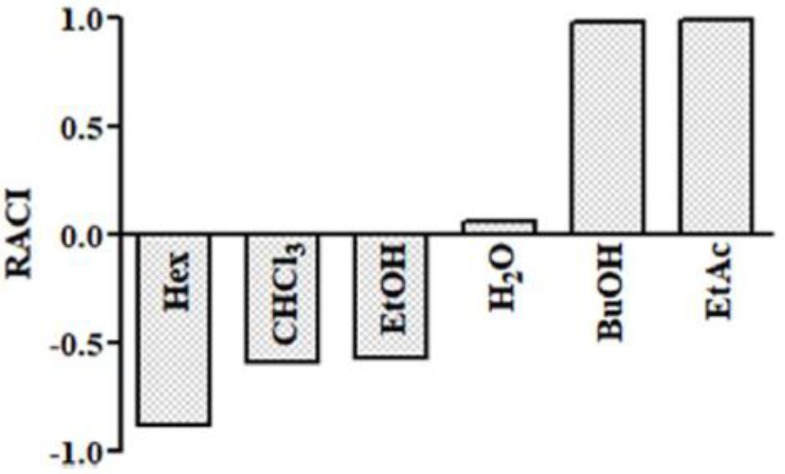
Relative antioxidant capacity index (RACI) of AG samples. Samples are ethanol extract (EtOH) and *n*-hexane (Hex), chloroform (CHCl_3_), ethyl acetate (EtAc), *n*-butanol (BuOH), and water (H_2_O) fractions of AG aerial parts.

The results obtained by ABTS, SO, and NO assays together with TPC have been integrated with each other by calculating the relative antioxidant capacity index (RACI) [39]. The EtAc and BuOH fractions had the highest RACI values (1.00 and 0.98, respectively) (Figure 3). The Hex fraction showed the lowest index (−0.88). In particular, the RACI value seems to be related to the high TPC of the EtAc and BuOH fractions.

### 2.3. Viability Analysis of MM and Healthy Cells Treated with AG Samples

**Figure 4:**
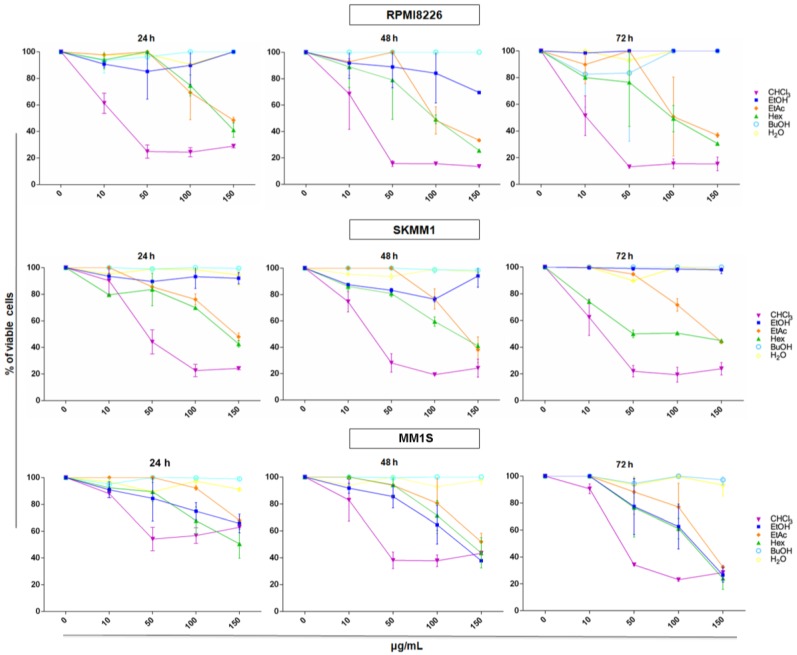
Viability assay of RPMI8226, SKMM1, and MM1S cells after treatment with AG samples at different concentrations (10, 50, 100, and 150 μg/mL) for 24, 48, and 72 h. Results are expressed as percent of cell viability normalized to DMSO-treated control cells. The line-graphs represent the average with standard deviation from three independent experiments. Samples are ethanol extract (EtOH) and *n*-hexane (Hex), chloroform (CHCl_3_), ethyl acetate (EtAc), *n*-butanol (BuOH), and water (H_2_O) fractions of AG aerial parts.

We treated MM cell lines, RPMI8226, SKMM1, and MM1S, with six different AG samples or with dimethyl sulfoxide (DMSO) vehicle control at different concentrations (10–150 μg/mL) for 24, 48, and 72 h (Figure 4). Viability test showed that most of samples, except for the EtOH extract, BuOH, and H_2_O fractions, exhibited a dose and time dependent anti-proliferative effect on MM cells. In particular, AG CHCl_3_ fraction was the most active one on all MM cell lines and its effect was observed already at 24 h of treatment with the lowest concentration (10 μg/mL).

We also calculated the concentration of the CHCl_3_ fraction that inhibited MM cell growth of 50% (EC_50_; Table 2). EC_50_ value was reduced over the time in all the MM tested lines. After 24 h of treatment, we obtained EC_50_ values of 16.74, 44.76, and 165.90 μg/mL for RPMI8226, SKMM1, and MM1S cells, respectively.

**Figure 5:**
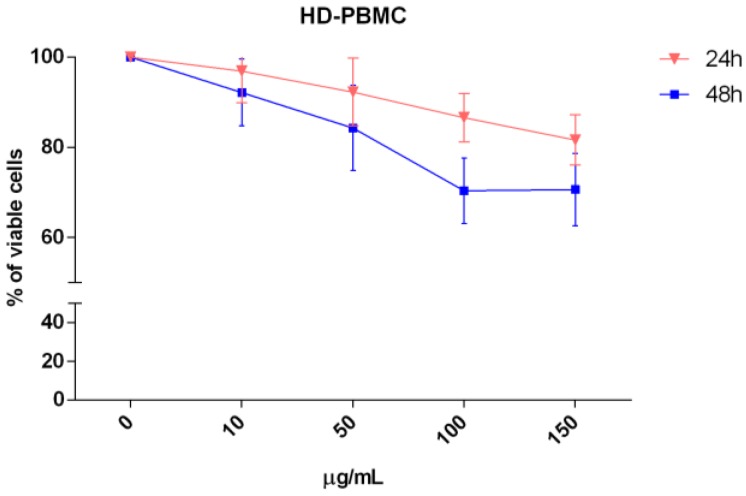
Analysis of five HD-PBMC viability after treatment with different concentrations (10, 50, 100, and 150 μg/mL) of AG CHCl_3_ fraction for 24 and 48 h. Results are expressed as percent of cell viability normalized to DMSO-treated control cells. The line-graphs represent the average with standard deviation from five healthy subjects.

Furthermore, to exclude any toxic effects on healthy cells, we treated peripheral blood mononuclear cells isolated from five healthy donors (HD-PBMCs) with different concentrations (10–150 μg/mL) of AG CHCl_3_ fraction for 24 and 48 h. Interestingly, the AG CHCl_3_ fraction had negligible effect on HD-PBMCs (Figure 5).

### 2.4. Evaluation of Apoptosis in MM Cells Treated with AG CHCl_3_ Fraction

**Figure 6:**
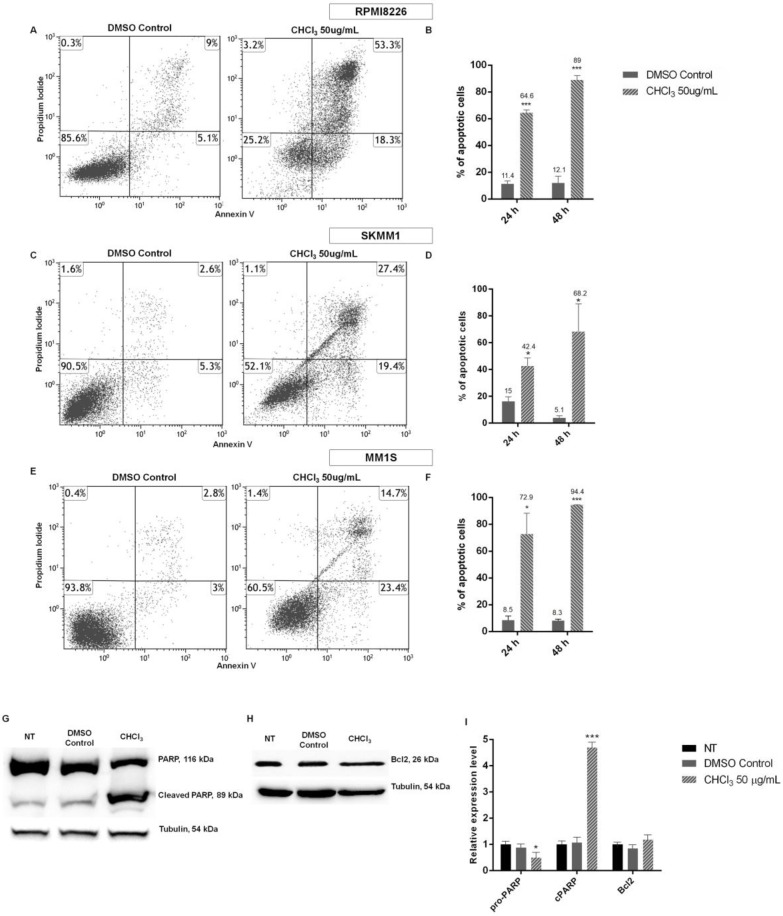
Cytofluorimetric evaluation of apoptosis/necrosis of RPMI8226 (**A**,**B**), SKMM1 (**C**,**D**), and MM1S (**E**,**F**) cell lines, after treatment with 50 μg/mL of AG CHCl_3_ fraction for 24 and 48 h. Dot plots (**A**,**C**,**E**) show a single representative experiment after 24 h of treatment; the bar-graphs (**B**,**D**,**F**) represent the average of percent of apoptosis, obtained from the sum of early and late apoptosis, of three independent experiments with standard deviation (* *p* < 0.05, *** *p* < 0.001). Western blot analysis of the AG CHCl_3_ fraction on the expression of PARP-1 (**G**), cleaved PARP (**G**), and Bcl2 (**H**) in RPMI8226 cells treated with 50 μg/mL of AG CHCl_3_ fraction for 24 h. Tubulin was used as a protein loading control. The bar-graphs (**I**) are representative of three independent experiments. Statistical analysis was carried out by a paired two-tailed Student’s *t*-test (* *p* < 0.05, *** *p* < 0.001, *n* = 3). Data were represented as mean ± standard error of the mean.

To investigate the anti-proliferative effect induced in cells treated with the AG CHCl_3_ fraction, we performed the apoptosis assay on MM cells using a concentration of 50 μg/mL. The AG CHCl_3_ fraction treatment for 24 and 48 h induced a significant increase of apoptosis in MM cells with respect to their control in a time dependent manner (Figure 6A–F). In particular, the percentage of apoptotic cells increased from 53.19% at 24 h in the CHCl_3_ fraction treated cells with respect to DMSO control to 76.99% at 48 h for RPMI8226 cells (Figure 6A,B), from 31.80% at 24 h to 63.15% at 48 h for SKMM1 cells (Figure 6C,D), and from 64.35% at 24 h to 86.25% at 48 h for MM1S cells (Figure 6E,F).

Moreover, apoptosis was also evaluated by western blot analysis in RPMI8226 cells (Figure 6G,I). In particular, the presence of the cleaved form of the caspase-3 substrate, the poly-ADP ribose polymerase (cleaved PARP-1), and the expression level of Bcl-2 were examined. We observed a significant activation of caspase-3 in MM cells treated for 24 h with 50 μg/mL of AG CHCl_3_ fraction compared to the control, detectable by the increase of cleaved PARP-1 (Figure 6G). Instead, the expression of Bcl-2 was the same in MM treated and control cells (Figure 6H).

### 2.5. Cell Cycle Analysis in MM Cells Treated with AG CHCl_3_ Fraction

**Figure 7:**
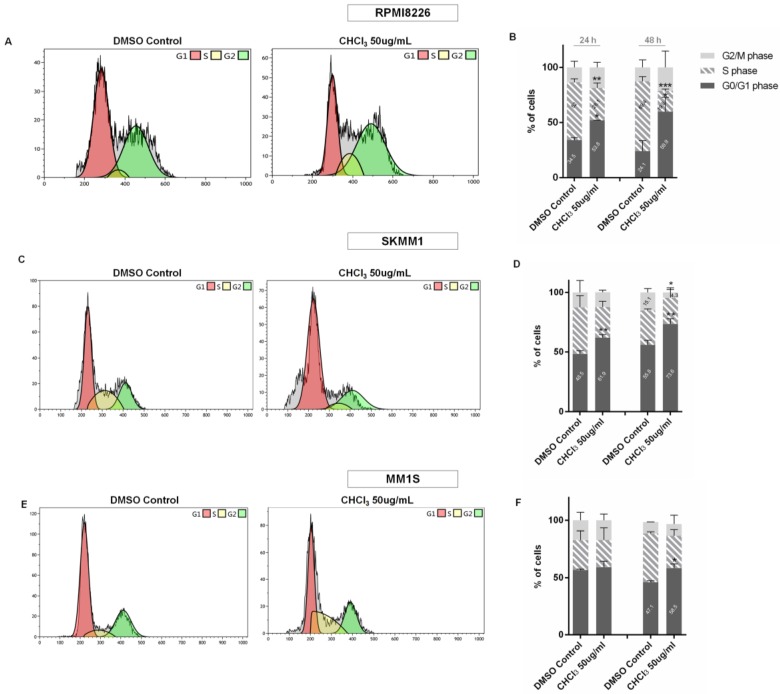
Cytofluorimetric evaluation of cell cycle on RPMI8226, SKMM1, and MM1S cell lines, at 50 μg/mL of AG CHCl_3_ fraction for 24 and 48 h. Cell cycle histograms (**A**,**C**,**E**) show a single representative experiment after 24 h of treatment; the bar-graphs (**B**,**D**,**F**) represent the average of three independent experiments with standard deviation (* *p* < 0.05, ** *p* < 0.01, *** *p* < 0.001).

Cell cycle cytofluorimetric analysis of MM cells treated with 50 μg/mL of AG CHCl_3_ fraction showed a G0/G1-phase arrest with respect to control in a time dependent manner (Figure 7B,D,F). In particular, the number of RPMI8226 treated cells in the G0/G1 phase significantly increased from 34.50% to 53.82% and from 24.00% to 60.00% after 24 and 48 h, respectively.

Moreover, a significant decrease of RPMI8226 treated cells in the S phase was observed at 24 and 48 h (28.1% and 19.8%, respectively) (Figure 7A,B).

A similar result was observed for SKMM1 cells: The percentage of cells in the G0/G1 phase increased from 48.46% to 61.88% and from 55.83% to 73.55% after 24 and 48 h of treatment, respectively.

In addition, after 48 h of treatment, the number of cells at the G2/M phase decreased from 15.10% to 4.34% for the control and SKMM1 cells, respectively (Figure 7C,D). For MM1S, we observed a significant increase of cells in the G0/G1 phase from 47.08% to 58.50% (*p* < 0.05) after 48 h of treatment (Figure 7E,F).

### 2.6. Cell Migration Assay in RPMI8226 Cells Treated with AG CHCl_3_ Fraction

**Figure 8:**
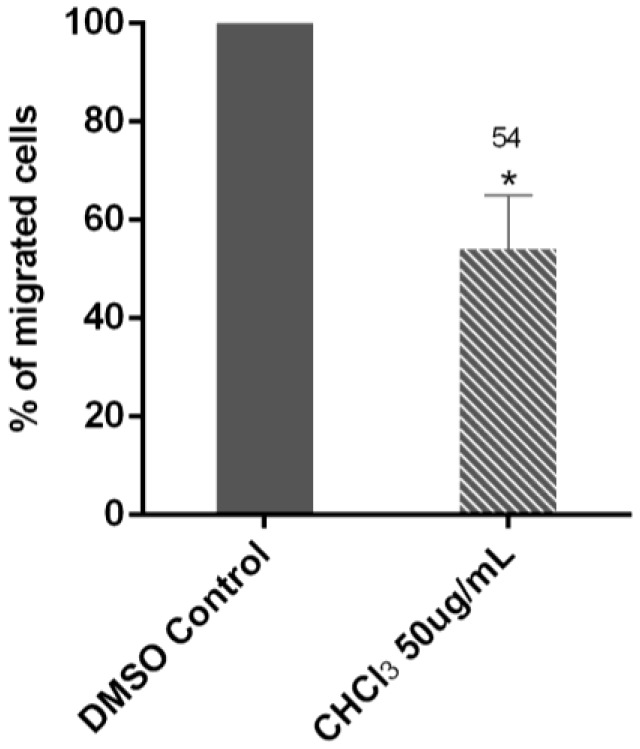
Transwell migration assays of RPMI8226 cells performed after treatment with 50 μg/mL of AG CHCl_3_ fraction and with DMSO control. Migrated cells were counted after 24 h of incubation. The bar-graphs represent the average of three independent experiments with standard deviation (* *p* < 0.05).

The effects of the AG CHCl_3_ fraction on the MM cell migration were investigated in RPMI8226 cells using a Transwell assay. Data showed that the AG CHCl_3_ fraction reduced the cell migration rate. The percentage of migrated RPMI8226 treated cells was 54% with respect to the DMSO control, indicating that AG CHCl_3_ induced a significant decrease of MM cell migration (*p* = 0.01) (Figure 8).

### 2.7. ROS Production and Mitochondria Membrane Potential (ΔΨ_m_)

**Figure 9:**
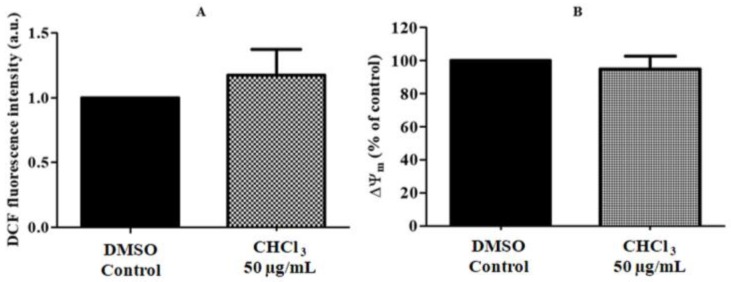
Evaluation of the intracellular ROS levels (**A**) and of ΔΨ_m_ depolarization (**B**) of RPMI8226 cells treated with 50 μg/mL of AG CHCl_3_ fraction. The values are means ± standard error of three replicates from three independent experiments.

The effects of the AG CHCl_3_ fraction on the intracellular redox status were investigated in RPMI8226 cells by the determination of the levels of ROS production. Data showed that the AG CHCl_3_ fraction did not significantly affect ROS formation at 50 µg/mL as compared with control cells (Figure 9A).

The Δ*Ψ_m_* was evaluated by the cation fluorescent probe, tetramethylrhodamine methyl ester (TMRM), to verify whether the ROS formation in RPMI8226 cells could be fitted with the changes or loss in Δ*Ψ_m_*. RPMI8226 cells exposed to 50 µg/mL of the AG CHCl_3_ fraction for 6 h did not show any significant difference compared to the control (Figure 9B).

## 3. Discussion

Natural products have been the cornerstones of anticancer pharmacology for many years and a significant number of compounds isolated from plants and microorganism are still tested nowadays for their anti-cancer activity [40,41,42,43,44], including hematologic malignancies [2,29,45].

In the present work, we suggest a sort of “future in the past” as we believe that, in the modern era of medicine engineering, natural-derived compounds, and plant extracts specifically, should continue to be considered one of the most important sources of natural and ideal anti-cancer drugs.

The biological activities of plant extracts are mainly due to their contents of polyphenols, flavonoids, and terpenoids. Numerous studies showed that these classes of compounds possess antioxidants, anti-inflammatory, and anticancer properties [21,24]. In this context, we reported that TPC, TFC, and TTeC of EtOH AG extract and its fractions differed among samples. These differences are congruent based on varying polarities of the used solvents. Fractions obtained with high polarity solvents reported the highest TPC and TFC, indicating that the majority of polyphenolic compounds in the AG samples could be polar compounds. At the same time, the highest total TTeC was reported in not polar CHCl_3_ and Hex fractions [46]. We found a correlation between TPC and antioxidant activity. Instead, TTeC was less involved in the scavenging activity.

The cytotoxic and apoptotic effects of different phyto-extracts on MM cells were recently documented in many studies [20,47,48,49]. In the present work, some fractions, except for the EtOH extract and BuOH and H_2_O fractions, showed a dose and time dependent anti-proliferative effect on the MM cells. Among all, AG CHCl_3_ and Hex fractions resulted in the most active. Probably, their cytotoxic effect could be due to the greater content of TTeC. In fact, AG CHCl_3_ contained more TTeC and, at the same time, showed a greater anti-proliferative effect compared to the Hex fraction. This result is in accordance with other in vitro and in vivo studies that reported terpenoids as inhibitors of both cell proliferation and tumor growth in several tumors, including breast, prostate, pancreatic carcinomas, lung cancer, and leukemia [24,50,51,52,53]. Furthermore, in a recent work, the cytotoxic activity of different Azorellane diterpenoids isolated from *A. compacta* on a breast cancer cell line (MCF-7) was described [54]. However, more studies are needed to investigate potential anti-proliferative effects of terpenoids in MM.

Interestingly, we observed an anti-proliferative effect of AG CHCl_3_ on three MM cell lines that harbor different chromosomal translocations [t(16;22), t(14;20) and t(14;16) for RPMI8226, SKMM1, and MM1S, respectively]. In particular, the translocation, t(14;16), represents a high-risk cytogenetic marker [55]. Our data agree with previous studies that described the cytotoxic activity on solid and haematological malignancies of other *Azorella* extracts. Sung et al. reported the cytotoxic activity of methanolic extract of *A. compacta* and its ability to induce apoptosis on leukemic HL60 cells [34]. Previously, another work described the antiproliferative activity of a new chalcone from *Azorella madreporica* on colon, breast, and prostate cancer cells [56]. All these data indicate the *Azorella* genus as a possible source of natural agent against different cancer types, including hematologic malignancies.

The main criterion for an “ideal” anti-tumor agent is the specificity of the action on tumor cells, with no or minimal toxicity on healthy ones. For this reason, in addition to efficacy, the safety profile for the development of a new anti-tumor agent should be taken into consideration. To our knowledge, few studies have verified plant extracts’ toxicity on healthy cells in a preliminary step. Interestingly, in the present work, we showed that AG CHCl_3_ fraction did not show toxicity towards the normal cells, like PBMCs.

Wong et al. reported that EC_50_ values are widely used to assess the potency of a compound [57]. Our data indicated that the EC_50_ value was reduced over the time in all the MM cells, with a mean of 22.06 ± 15.56 μg/mL after 72 h of treatment. These data are in agreement with a report demonstrating that a natural extract possessing an EC_50_ value of about 20 μg/mL is considered to have anti-cancer therapeutic value [58]. Thus, the fact that our AG CHCl_3_ fraction showed an EC_50_ value around 20 μg/mL encourages further investigation for its possible therapeutic application.

Another essential characteristic of an antitumor drug is the ability to induce tumor cell apoptosis. In fact, the simultaneous ability of a substance to inhibit cell growth and to induce apoptosis allows discrimination between anti-tumor agents and toxic ones [34]. Our data showed a significant increase of apoptotic cells during treatment with the AG CHCl_3_ fraction. Several works have demonstrated that the block in the G0/G1 phase is a characteristic feature of apoptosis [59,60,61,62]. Our data showed an increase of treated cells in the G0/G1 phase in a time dependent manner, with a corresponding decline of cells in the S and G2/M phases, confirming apoptosis. In addition, the AG CHCl_3_ fraction induced a strong inhibition of migration in myeloma cells, however, apoptosis was promoted.

Furthermore, to get a preliminary indication of the cellular mechanisms by which the AG CHCl_3_ fraction induced cell death, the expression level of apoptosis-related proteins was detected by western blot analysis. AG CHCl_3_ fraction-treated cells showed an increase of PARP-1 cleavage, but no reduction of Bcl-2 protein levels were observed. These results indicated that, probably, the death process was not regulated through a mitochondrial damaged characteristic of the intrinsic pathway. In fact, both the extrinsic and intrinsic apoptotic pathways may be involved in caspase-3 cleavage [63], but Bcl-2 is implicated only in the intrinsic pathways. Bcl-2 blocks the release of cytochrome c from the mitochondria into the cytosol [64], inhibiting changes in mitochondrial membrane potential and mitochondrial permeability [65].

Accumulation of ROS and the disruption of ΔΨ_m_ represents an early event in the intrinsic pathway of apoptosis [66]. In our setting, the AG CHCl_3_ fraction did not significantly stimulate ROS formation in treated MM cells as compared with the control. ROS concentration was also not reduced. These results were the same that have been shown using in vitro assays in which we did not observe a strong radical scavenging activity of the AG CHCl_3_ fraction. In addition, no loss of ΔΨ_m_ was detected in MM cells treated with the AG CHCl_3_ fraction, pointing out that AG CHCl_3_ fraction-induced apoptosis could not be regulated through mitochondrial damage.

In summary, our data strongly shows that AG CHCl_3_ fraction has in vitro potential with anti-MM effects compared to HD-PBMCs, giving an additional advantage over other fractions. Considering the lack of literature knowledge on the anticancer effects of AG samples, this study represents pioneering research in this area. Further investigations are ongoing to (i) identify specific bioactive compounds; (ii) investigate the molecular mechanisms underlying anticancer activity; and (iii) define how these natural extracts could be used as a complementary approach to current therapies for MM. Altogether, our data indicate that AG samples could represent a promising source of natural anti-MM compounds.

## 4. Materials and Methods

### 4.1. Chemicals and Reagents

Fetal bovine serum (FBS), RPMI 1640, phosphate-buffered saline (PBS), and penicillin/streptomycin were purchased from Gibco-BRL (Life technologies, Carlsbad, CA, USA).

Solvents as ethanol, *n*-hexane, chloroform, ethyl acetate, *n*-butanol, methanol, sulfuric acid, and hydrochloric acid were purchased from Carlo Erba (Milano-Italy).

Folin-Ciocalteu reagent 2N, sodium carbonate, sodium nitrate, aluminum chloride, sodium hydroxide, 2.2′-azino-bis (3-ethylbenzothiazoline-6-sulfonic acid) (ABTS), dimethyl sulfoxide (DMSO), potassium persulfate, potassium phosphate monobasic, *β*-nicotinamide adenine dinucleotide reduced form (NADH), phenazinemethosulfate (PMS), nitrotetrazolium blue chloride (NBT), sodium nitroprusside dehydrate (SNP), sulfanilamide, *N*-(1-Naphthyl) ethylenediaminedihydrochloride, sodium acetate trihydrate, 2′,7′-dichlorodihydrofluorescein diacetate (DCFH-DA), Tris (hydroxymethyl) aminomethane (Tris),sodium chloride, tergitol™ solution (NP40), sodium dodecyl sulfate (SDS), NFM, TBST, sodium deoxycholateand standards as gallic acid, quercetin, linalool, 6-hydroxy-2.5.7.8-tetramethylchroman-2-carboxylic acid (Trolox), and anti-rabbit secondary antibody were purchased from Sigma-Aldrich (Milano, Italy).

A cellTiter 96 Aqueous One Solution assay kit (MTS) was purchased from Promega (Madison, WI, USA).

Nitrocellulose membrane was purchased from GE Healthcare (Chicago, IL, USA).

A FITC Annexin V Apoptosis Detection kit was purchased from Becton Dickinson (BD Pharmingen, San Jose, CA, USA).

24-well Millicell Hanging cell culture transwell inserts 8 µm PET (Millipore Corporation, Billerica, MA, USA).

TMRM (Life Technologies, Carlsbad, CA, USA) was a kind gift from Dr. M. Lasorsa (IBBE, CNR, Bari).

Antibody anti-PARP-1 was purchased from CST (Danvers, MA, USA).

Antibody anti-Bcl-2 and chemiluminescence (ECL plus) kit were purchased from Thermo Fisher (Rodano, Milano, Italy).

### 4.2. Preparation of AG Samples

The AG aerial parts were collected in Bolivia near the Aymaya population/community (18.45° S to 66.46° W; 3750 msnm), Bustillo province, Potosí department, Bolivia. Samples of the species are found in the herbal medicinal plants of the National University Siglo XX, Llallagua, Potosí, Bolivia.

A voucher specimen was stored at the University of La Paz, dried at room temperature, crushed, and extracted by dynamic maceration with 96% EtOH (solid to solvent ratio of 1:10 *w*/*v*). The EtOH extract was filtrated through a 0.45 µm Buchner funnel and dried by a rotary evaporator. Then, 22.00 g of EtOH extract were solved in 220 mL of distilled H_2_O and subjected to liquid/liquid extraction with *n*-hexane, chloroform, ethyl acetate, and *n*-butanol solvents to make a chemical compound separation.

### 4.3. Healthy Donors, MM Cell Lines and Chemicals

Five healthy subjects gave informed consent. Peripheral blood was drawn into EDTA tubes and PBMCs were collected by Ficoll-hypaque gradient separation. MM cell lines, SKMM1, RPMI8226, and MM1S, were purchased from American Type Culture Collection (ATCC) and cultured in RPMI 1640 supplemented with 10% FBS and 1% of penicillin/streptomycin. All cell lines were grown at 37 °C in 5% CO_2_. All AG samples were dissolved in DMSO at the stock solution of 30 mg/mL and then diluted in FBS for cell treatments. The final DMSO concentration in the cultures was no greater than 0.5%.

### 4.4. Total Polyphenol Content (TPC), Total Flavonoid Content (TFC), and Total Terpenoid Content (TTeC)

The dried crude EtOH extract and Hex, CHCl_3_, EtAc, and BuOH and H_2_O fractions were tested for their TPC, TFC, and TTeC content. The Folin-Ciocalteu assay as reported by Todaro et al. (2017) with slight modifications was used to determine the TPC content of AG samples [67]. The results were expressed as mg of gallic acid equivalents per gram of dried sample (mg GAE/g).

The TFC was determined using a solution with 5% NaNO_3_, 1% AlCl_3_, and 1 M NaOH solution [38]. The results were expressed as mg of quercetin equivalents per gram of dried sample (mg QE/g) after the absorbance measurement at 510 nm.

The evaluation of TTeC was performed by a rapid and high-throughput assay using the monoterpene linalool as the standard reagent. The absorbance was measured at 538 nm and the results were expressed as mg of linalool equivalents per gram of dried sample (mg LE/g).

### 4.5. Radical-Scavenging Activity

The antioxidant activity of AG samples was tested by three different in vitro antioxidant tests. In particular, we determined the radical scavenging activity by synthetic ABTS assay and physiological superoxide anion (SO) and nitric oxide (NO) radicals. The synthetic ABTS^·+^ radical and the biological SO and NO radicals were generated in different experiments and the capacity of the samples to scavenge these radicals was monitored by spectrophotometer (SPECTROstar^Nano^, BMG Labtech) [68,69].

#### 4.5.1. ABTS Assay

The 2.2′-azinobis-(3-ethylbenzothiazoline-6-sulfonic acid) diammonium salt (ABTS) radical assay was used to determine the radical-scavenging capacity of AG samples [70] against the ABTS^·+^ radical.

#### 4.5.2. Super Oxide (SO) Anion Scavenging Activity

The inhibition of formazan formation from samples was monitored at 560 nm and the results were expressed as the concentration inhibiting 25% of radical inhibition in mg/mL (IC_25_). The ascorbic acid was used as the positive control [38].

#### 4.5.3. Nitric Oxide (NO) Radical Scavenging Activity

The nitric oxide interacts with oxygen to give nitrite ions that can be determined spectrophotometrically by Griess reagent [38]. Results were expressed as IC_25_ of radical inhibition in mg/mL and ascorbic acid was used as the positive control.

### 4.6. Cell Viability Assay

Cell viability was assessed using the MTS assay [71,72]. In brief, cells were seeded into 96-well plates (3 × 10^4^ cell/100 µL medium), treated with AG samples at different concentrations (10, 50, 100, and 150 µg/mL), and incubated for 24, 48, and 72 h. The optical density was measured at 492 nm. All experiments were conducted in triplicate. Cell viability was calculated as the percentage of MM viable cells in AG vs DMSO treated cells. The EC_50_ was determined by GraphPad Prism (GraphPad Prism, San Diego, CA, USA).

### 4.7. Apoptosis Assay

Apoptotic cells were detected by cytometric analysis using a FITC Annexin V Apoptosis Detection kit (BD Pharmingen), according to the manufacturer’s protocol. Briefly, MM cells were plated in a 6-well culture plate at a density of 4 × 10^5^ cell/well, treated with 50 µg/mL of AG CHCl_3_ fraction, and incubated for 24 and 48 h. After treatment, cells were harvested, washed, and resuspended in Annexin V binding buffer [73]. Next, cells were labeled with 5 µL of FITC Annexin V and 5 µL of propidium iodide (PI). Stained cells were incubated at dark for 15 min. 1 × 10^4^ events were acquired using a NAVIOS flow cytometer and analyzed by Kaluza 2.0 software (Beckman Coulter, Life Sciences, Indianapolis, IN, USA). Both single positive for Annexin V and double positive for Annexin V and PI cells were interpreted as signs of early and late phases of apoptosis, respectively.

### 4.8. Cell Cycle Analysis

After treatment with 50 µg/mL of AG CHCl_3_ fraction for 24 and 48 h, MM cells were harvested, washed, and fixed in cold ethanol 70% for 1 h. Fixed cells were then labeled with PI/RNase staining solution for 30 min at room temperature in the dark [73,74]. A total of 1 × 10^4^ events were acquired by NAVIOS flow cytometer and analyzed by Kaluza 2.0 software (Beckman Coulter, Life Sciences, Indianapolis, IN, USA).

### 4.9. Cell Migration Assays

Transwell inserts with 8 μm pores were inserted into a 24-well plates for migration studies. 1 × 10^5^ RPMI8226 cells treated with 50 µg/mL of AG CHCl_3_ fraction and with DMSO control were seeded into the inserts in 300 μL of serum-free RPMI 1640 medium. 500 μL of RPMI 1640 medium with 10% FBS were placed in the single well of a 24 well plate. After 24 h of incubation, cells that migrated into the lower chamber were counted. Triplicates of each experiment were performed.

### 4.10. Measurement of Reactive Oxygen Species (ROS) Generation and of Mitochondrial Membrane Potential (ΔΨ_m_)

2.5 × 10^5^ RPMI8226 cells were treated with 50 µg/mL of AG CHCl_3_ fraction for 6 h and used to measure ROS and ΔΨ_m_ values, as reported in the following paragraphs.

#### 4.10.1. ROS Generation

The level of intracellular ROS was determined following the method reported in Armentano et al. (2015) with slight modification [70]. Briefly, RPMI8226 cells were stained with 10 µM of DCFH-DA and incubated for 30 min at 37 °C in the dark. The fluorescence was measured by FACSCanto II flow cytometer and data were analyzed by DIVA software (BD Biosciences, San Jose, CA, USA).

#### 4.10.2. ΔΨ_m_ Measurement

The level of ΔΨ_m_ was monitored by flow cytometry as reported by Armentano et al. (2015) [70]. In brief, cells were incubated with 150 nM of TMRM in PBS for 20 min at 37 °C in the dark. Then, cells were analysed by FACSCanto II flow cytometer and data were analyzed by DIVA software.

### 4.11. Western Blot Analysis

A total of 2 × 10^6^ of untreated, DMSO, and 50 μg/mL of AG CHCl_3_-treated RPMI8226 cells were collected and lysed in buffer (50 mM Tris pH 7.4, 150 mMNaCl, 1% NP40, 0.1% SDS, 0.5% sodium deoxycholate) supplemented with protease inhibitors cocktail (Sigma Aldrich, Milano, Italy), followed by centrifugation at 13,000× *g* for 30 min at 4 °C. The protein concentration in each sample was detected using the Bradford assay. Equal amounts of sample lysate (80 μg) were separated by SDS-PAGE (4–8% for PARP and 4–15% for Bcl2) and transferred to nitrocellulose membrane. Nonspecific binding sites were blocked with TBST buffer containing 5% nonfat dry milk (NFM) at room temperature for 1 h. Subsequently, membranes were incubated with primary antibodies anti-PARP-1 (1:1000 in 5% NFM/TBST) and anti-Bcl-2 (1:200 in 5% NFM/TBST) overnight at 4 °C. After incubation with HRP-conjugated anti-rabbit secondary antibody (1:10,000), detection was performed using the enhanced chemiluminescence (ECL plus) kit.

### 4.12. Statistical Analysis

All data were expressed as mean ± standard deviation or mean ± standard error of mean of three independent experiments. To verify the correlation among the used methods, the *p* values were analysed by one-way analysis of variance (ANOVA); a Pearson coefficient and *T*-test were determined using GraphPad Prism 5 Software (San Diego, CA, USA).

## Figures and Tables

**Table 1:** Results of ABTS, super oxide (SO), and nitric oxide (NO) scavenging activity of AG samples.

Samples	ABTS (mgTE/g)	SO (IC_25_ mg/mL)	NO (IC_25_ mg/mL)
EtOH extract	76.83 ± 1.23 ^a^	2.59 ± 0.11 ^a^	/
Hex fraction	/	/	/
CHCl_3_ fraction	32.08 ± 0.02 ^b^	0.47 ± 0.02 ^b^	/
EtAc fraction	282.26 ± 9.53 ^c^	0.12 ± 0.01 ^c^	/
BuOH fraction	206.65 ± 7.28 ^d^	0.20 ± 0.01 ^c^	9.13 ± 0.09 ^a^
H_2_O fraction	65.09 ± 0.40 ^a^	0.37 ± 0.02 ^b^	8.94 ± 0.07 ^b^

Data are expressed as means ± standard deviation from three experiments; mg TE/g = mg of trolox equivalents per gram of dried sample; IC_25_ mg/mL = concentration of the samples required to inhibit the activity of the radical by 25%; different superscripts in the same row indicate significant difference (*p* < 0.05); / = below the detection limit of the assay. Samples are ethanol extract (EtOH) and n-hexane (Hex), chloroform (CHCl_3_), ethyl acetate (EtAc), n-butanol (BuOH), and water (H_2_O) fractions of AG aerial parts.

**Table 2:** EC_50_ values of the AG CHCl_3_ fraction on MM cells.

MM Cell Lines	24 h μg/mL	48 h μg/mL	72 h μg/mL
RPMI8226	16.74	17.38	10.03
SKMM1	44.76	25.75	16.52
MM1S	165.90	53.02	39.63
